# α-Spinasterol, a TRPV1 receptor antagonist, elevates the seizure threshold in three acute seizure tests in mice

**DOI:** 10.1007/s00702-015-1391-7

**Published:** 2015-03-13

**Authors:** Katarzyna Socała, Dorota Nieoczym, Mateusz Pieróg, Piotr Wlaź

**Affiliations:** Department of Animal Physiology, Institute of Biology and Biochemistry, Faculty of Biology and Biotechnology, Maria Curie-Skłodowska University, Akademicka 19, PL 20-033 Lublin, Poland

**Keywords:** α-spinasterol, TRPV1 receptors, Anticonvulsant activity, Seizure threshold, Mice

## Abstract

α-Spinasterol is a plant-derived compound which was reported to act as a selective antagonist for the transient receptor potential vanilloid 1 (TRPV1) receptor. Several studies revealed that the TRPV1 receptors might modulate seizure activity in animal models of seizures and epilepsy. The aim of the present study was to investigate the effect of α-spinasterol on the seizure threshold in three acute models of seizures, i.e., in the intravenous (i.v.) pentylenetetrazole (PTZ) seizure test, in the maximal electroshock seizure threshold (MEST) test and in the model of psychomotor seizures induced by 6 Hz stimulation in mice. Our results revealed significant anticonvulsant effect of α-spinasterol in all the used seizure tests. In the i.v. PTZ test, statistically significant elevation was noted in case of the threshold for myoclonic twitches (doses of 0.1–1 mg/kg) and generalized clonus seizures (doses of 0.5 and 1 mg/kg) but not for tonic seizures. The studied TRPV1 antagonist also increased the threshold for tonic hindlimb extension in the MEST (doses of 0.5 and 1 mg/kg) and 6 Hz psychomotor seizure (doses of 0.1 and 0.5 mg/kg) tests in mice. Furthermore, α-spinasterol did not produce any significant impairment of motor coordination (assessed in the chimney test) and muscular strength (investigated in the grip-strength test) and it did not provoke significant changes in body temperature in mice. Based on the results of our study and the fact that α-spinasterol is characterized by good blood–brain permeability, we postulate further investigation of this compound to precisely evaluate mechanism of its anticonvulsant action and opportunity of its usage in clinical practice.

## Introduction

The transient receptor potential vanilloid 1 receptor (TRPV1), first identified in 1997 as a capsaicin receptor (Caterina et al. 1997), is a non-selective cation channel composed of six transmembrane domains with a short cation-permeable pore region (Pingle et al. 2007; Vriens et al. 2009). TRPV1 is a polymodal receptor, capable of responding to a wide range of stimuli such as noxious heat (>43 °C), voltage, protons/cations, various natural compounds (capsaicin, resiniferatoxin, piperine) and endovanilloids which act as endogenous agonists (anandamide, arachidonic acid metabolites and polyamines). The TRPV1 receptors are predominantly expressed in peripheral sensory neurons, particularly in nociceptive Aδ and C fibres, where they play a crucial role in temperature sensing and noxious stimuli detection (Szallasi et al. 2007; Vriens et al. 2009). Numerous studies showed their importance in mediating various pain conditions, especially inflammatory thermal hyperalgesia and chronic pain. Therefore, TRPV1 receptors have been the most extensively studied as a molecular target for pain relief (Szallasi et al. 2007).

The distribution of the TRPV1 receptors is not limited only to the peripheral nervous systems. Their presence was also reported in the brain structures including hypothalamus, cerebellum, cerebral cortex, midbrain, hippocampus and amygdala (Premkumar and Sikand 2008; Martins et al. 2014). The TRPV1 receptors in peripheral neurons are targeted mainly by noxious or thermal stimuli. In contrast, the TRPV1 receptors in the brain are likely activated by endogenous agonists (Martins et al. 2014). It seems that the TRPV1 receptors play multiple functions within the brain including neurotransmission and synaptic plasticity (Kauer and Gibson 2009). Several studies showed that the TRPV1 receptors may be implicated in the neurobiology of addictive behavior, anxiety, mood disorders, cognition and emotion (Manna and Umathe 2012a; Martins et al. 2014).

A growing number of recent studies indicate the involvement of the TRPV1 receptors in the etiopathogenesis of epilepsy. Overexpression of the TRPV1 receptors was reported in the dentate gyrus of mice after pilocarpine-induced status epilepticus (Bhaskaran and Smith 2010) as well as in the temporal cortex and hippocampus from patients with mesial temporal lobe epilepsy (Sun et al. 2013). Furthermore, Gonzalez-Reyes et al. (2013) reported that the blockage of the TRPV1 receptors with capsazepine suppresses the seizure activity induced by 4-aminopyridine in mice and epileptiform activity in vitro. In a more recent study, *N*-oleoyldopamine (a TRPV1 receptor agonist) accelerated the pentylenetetrazole (PTZ) kindling in rats, while AMG-9810 (a TRPV1 receptor antagonist) delayed the development of seizure following repeated administration of PTZ. Likewise, in amygdala kindling in rats, *N*-oleoyldopamine and AMG-9810 exerted anti- and pro-convulsant effects, respectively (Shirazi et al. 2014). Thus, it was suggested that the inactivation of the TRPV1 receptors may offer a new therapeutic approach in the treatment of epilepsy (Fu et al. 2009; Gonzalez-Reyes et al. 2013).

Brain penetration of the TRPV1 receptor antagonists is a critical feature that is highly desirable for studying their effects on seizure activity. Poor blood–brain barrier permeability is an obstacle for systemic administration of some commercially available TRPV1 antagonists. The second drawback of many TRPV1 antagonists is an induction of severe hyperthermia (Premkumar and Sikand 2008; Trevisan et al. 2012). α-Spinasterol, also known as bessisterol, hitodesterol or α-spinasterin, is a phytosterol first isolated from the spinach leaves and alfalfa (Nilius and Appendino 2013) that has been reported to possess antinociceptive, anti-inflammatory, antitumor, antiulcerogenic and cytoprotective properties (Jeon et al. 2005; Jeong et al. 2010; Trevisan et al. 2012; Klein-Junior et al. 2012; Borges et al. 2014). Notably, α-spinasterol was reported to act as a selective TRPV1 antagonist with high penetration into the brain after systemic administration and it is devoid of side effects such as hyperthermia (Trevisan et al. 2012). These features make α-spinasterol a useful tool for studying the role of the TRPV1 receptors in neurobiology of seizures.

Therefore, the present study was undertaken to evaluate the effect of α-spinasterol on the seizure thresholds in three acute seizure tests in mice with predictive validity of clinical efficacy that are recommended for the primary screen of antiepileptic drugs (Löscher 2011). Moreover, acute adverse effects of α-spinasterol on neuromuscular strength (the grip-strength test), motor coordination (the chimney test) and body temperature were investigated.

## Materials and methods

### Animals

All experiments were performed on naïve male Albino Swiss mice weighing 25–30 g. The animals were purchased from a licensed breeder (Laboratory Animals Breeding, Ilkowice, Poland) and housed in groups of 8–10 in Makrolon cages (37 cm × 21 cm × 14 cm) under strictly controlled laboratory conditions (temperature maintained at 22–23 °C, relative humidity about 45–55 %) with an artificial 12/12 h light/dark cycle (light on at 6:00 a.m.). A nutritionally balanced rodent chow diet (Agropol S.J., Motycz, Poland) and tap water were continuously available. Before being used in the experiments, mice were allowed an adaptation period of at least 7 days. All experiments were performed between 8:00 am to 2:00 p.m. to minimize circadian influences, after a minimum 30-min acclimatization to the experimental room.

The total number of animals used in the present study was 227. The animals were randomly assigned to the experimental groups as follows: 13–15 animals/group in the intravenous PTZ test, 18–21 animals/group in the MEST and 6 Hz seizure threshold test and 11–12 animals/group in the grip-strength test, the chimney test and for temperature measurement. The ‘up-and-down’ method for seizure threshold determination in the MEST and 6 Hz seizure tests requires ~20 animals per group because only one part of the group (with or without seizures) is used to calculate the seizure threshold. Since the grip-strength test, the chimney test and the temperature measurement are very quick and non-invasive procedures, all the tests performed in the same groups of animals shortly before the i.v. PTZ seizure test. This allowed to limit the total number of animals used in the present study, what was in accordance with the guidelines of the Ethical Committee.

The study was carried out under experimental protocols approved by the Ethical Committee of the Medical University in Lublin (license number 26/2014). All procedures were in strict compliance with the European Union Directive of 22 September 2010 (2010/63/EU) and Polish legislation concerning animal experimentation. All efforts were made to minimize animal suffering as well as the number of animals used in the study.

### Drugs

α-Spinasterol (5α-Stigmasta-7,22-dien-3β-ol) was purchased from TRC (Toronto Research Chemicals Inc., Canada), dissolved in a 1 % solution of dimethyl sulfoxide (DMSO, ICN Biomedicals, Inc., Aurora, OH, USA) in normal saline and administrated intraperitoneally (i.p.) in a constant volume of 10 ml/kg. Pretreatment time for α-spinasterol (30 min) was based on literature search (Klein-Junior et al. 2012; Borges et al. 2014). Control animals received i.p. injection of 1 % DMSO.

The dose of 0.1 mg/kg (in a volume of 10 ml per kg of body weight) was selected as an initial dose for all experiments. The dose was then increased or decreased depending on the observed results to see if there is a dose-dependent effect.

Pentylenetetrazole (PTZ; Sigma-Aldrich, Poznań, Poland) was dissolved in normal saline and infused intravenously (i.v.).

### PTZ-induced seizure threshold test

Thirty minutes following i.p. administration of α-spinasterol or vehicle, mice were placed in the cylindrical plastic restrainer (12-cm long, 3-cm inner diameter). The lateral tail vein was catheterized with a 2-cm-long 27-gauge needle attached by polyethylene tubing PE20RW (Plastics One Inc., Roanoke, VA, USA) to a 5-ml plastic syringe containing a 1 % solution of PTZ. The syringe was mounted on a syringe pump (model Physio 22, Hugo Sachs Elektronik–Harvard Apparatus GmbH, March-Hugstetten, Germany). After correct needle placement into the tail vein, which was verified by the appearance of blood in the tubing, mice were placed in a Plexiglas arena for behavioral observation. PTZ solution was infused at a constant rate of 0.2 ml/min. The time intervals from the start of PTZ infusion to the onset of the following endpoints were recorded: (1) the initial myoclonic twitch, (2) generalized clonus with loss of righting reflex and (3) tonic forelimb extension. The seizure thresholds were calculated separately for each endpoint according to the following formula: threshold dose of PTZ (mg/kg) = (infusion duration (s) × infusion rate (ml/s) × PTZ concentration (mg/ml) × 1000)/body weight kg. Seizure thresholds were expressed as the amount of PTZ (in mg per kg) ± SEM (standard error of the mean) needed to produce the first apparent sign of each endpoint in each experimental group. Tonic convulsions were usually lethal for mice. All surviving animals were euthanized immediately after the end of the infusion.

### Maximal electroshock seizure threshold (MEST) test

To determine the threshold for maximal electroshock seizures, constant current stimuli (50 Hz sine-wave, 0.2 s) were applied via saline-soaked transcorneal electrodes after application of ocular anesthetic with the usage of rodent shocker (type 221; Hugo Sachs Elektronik, Freiburg, Germany). A drop of ocular anesthetic (1 % solution of tetracaine hydrochloride, Sigma-Aldrich) was applied into each eye 1 min before stimulation. During stimulation mice were restrained manually and immediately following stimulation were placed in a Plexiglas arena (37 cm × 21 cm × 14 cm) for behavioral observation for the presence or absence of seizure activity. Tonic hindlimb extension was taken as an endpoint. The current intensity was established according to an ‘up-and-down’ method described by Kimball et al. (1957). Current intensity was lowered or raised by 0.06-log intervals depending on whether the previously stimulated animal did or did not exert tonic hindlimb extension, respectively. The data obtained in groups of 19–20 animals were used to determine the threshold current causing endpoint in 50 % of mice (CS_50_ with confidence limits for 95 % probability).

### Six-hertz (6 Hz) psychomotor seizure threshold test in mice

Psychomotor seizures were induced via corneal stimulation. Square-wave alternating current stimuli (0.2-ms duration pulses at 6 Hz for 3 s) were applied via saline-soaked corneal electrodes using a Grass S48 stimulator coupled with a constant current unit CCU1 (both from Grass Technologies, West Warwick, RI, USA). A drop of ocular anesthetic (1 % solution of tetracaine hydrochloride) was placed on the corneas before the stimulation and the electrodes were soaked in the 0.9 % saline immediately before testing to ensure a good electrical contact. Mice were restrained manually during electrical stimulation and immediately following stimulation, the animals were placed in a Plexiglas arena (37 cm × 21 cm × 14 cm) for behavioral observation. Psychomotor seizures were characterized by immobility or stun posture, jaw and forelimb clonus, twitching of the vibrissae and elevated or Straub tail (Barton et al. 2001). The lack of the features listed above or the resumption of normal exploratory behavior within 10 s after stimulation were considered indicators of the absence of seizures. The mice were subjected to stimuli with different current intensities according to an ‘up-and-down’ method (Kimball et al. 1957). Each animal was stimulated only once at any given current intensity that was lowered or raised by 0.06-log intervals depending on whether the previously stimulated animal did or did not respond with convulsions, respectively. The data obtained in groups of 18–21 animals were used to determine the threshold current causing 6 Hz-induced seizures in 50 % of mice (CS_50_ with confidence limits for 95 % probability).

### Grip-strength test

The acute adverse effect of α-spinasterol on skeletal muscular strength in mice (12 animals/group) was determined in the grip-strength test. The grip-strength apparatus (BioSeb, Chaville, France) consisted of a steel wire grid (8 × 8 cm) connected to an isometric force transducer. The mouse was lifted by its tail so that it could grasp the grid with its forepaws. The animal was then pulled back steadily by the tail until it released the grid. When the animal released the grid, the maximal grip-strength value (in newtons, N) of the animal was displayed on the screen. The procedure was repeated three times and the mean force exerted by each mouse before losing grip was recorded. Since body weight is a factor that affects the grip force, the mean force was normalized to body weight and expressed in mN/g ± SEM.

### Chimney test

The chimney test was used to assess the acute adverse effects of α-spinasterol on motor performance in mice (12 animals/group). In this test, the inability of an animal to climb backward up through a Plexiglas tube (inner diameter 3 cm, length 30 cm) within 60 s was an indication of motor impairment.

### Rectal temperature measurement

Variation in rectal temperature was recorded using an electronic thermometer (ThermoWorks, Alpine, Utah, USA) by inserting the rectal probe to a depth of ~2 cm into the rectum of the mouse. The rectal temperature of each animal was recorded just before administration of α-spinasterol and 30 min later. The difference between the pre-injection and post-injection values was then calculated and expressed as ΔT (°C).

### Statistical analysis

Statistical analysis was performed using one-way analysis of variance (one-way ANOVA) followed by the Dunnett’s post hoc test for multiple comparisons. To perform statistical analyses of data obtained in the MEST and the 6 Hz seizure tests, the CS_50_ values with 95 % confidence limits were transformed into the mean value of logarithms (of current strength) with standard deviation. The results from the chimney test were analyzed with the Fisher’s exact probability test. Statistical significance was noted when p values were equal to or less than 0.05. All calculations were carried out with GraphPad Prism version 5.03 for Windows (GraphPad Software, San Diego, CA, USA.).

## Results

### Effects of α-spinasterol in the PTZ-induced seizure threshold test

**Fig. 1:**
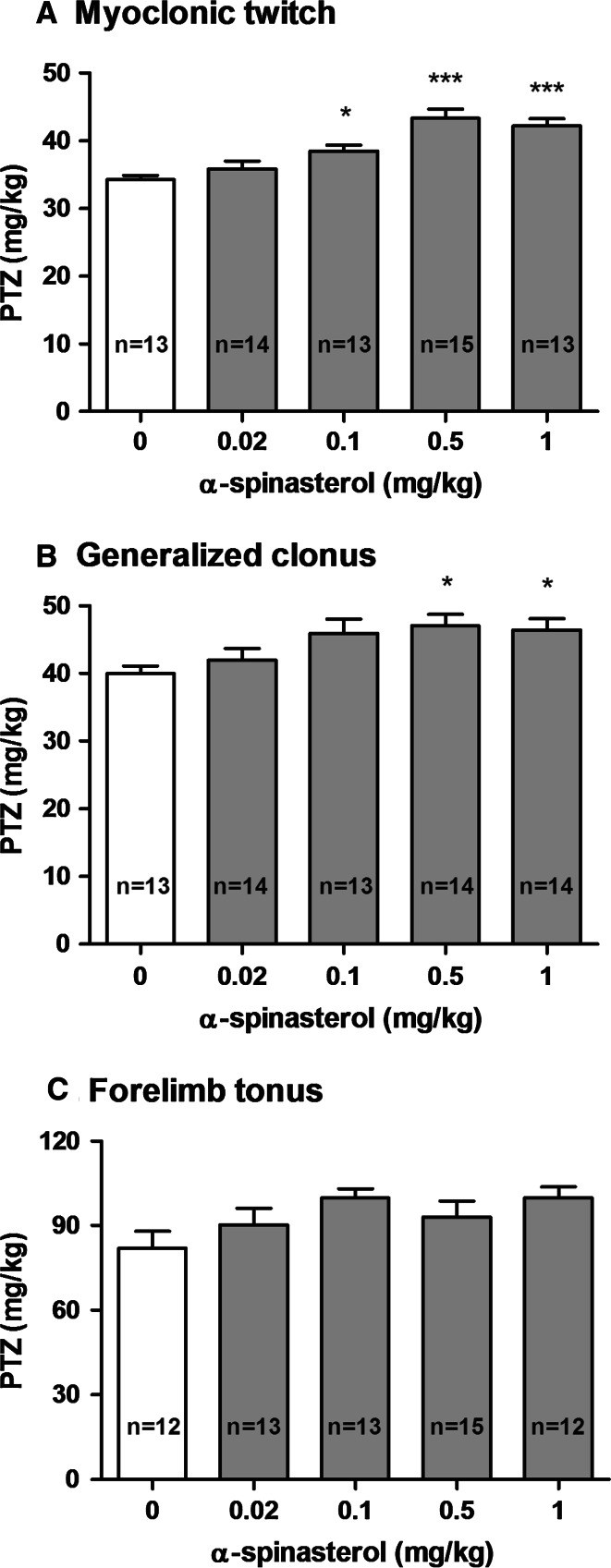
Effect of α-spinasterol on the threshold for the onset of first myoclonic twitch (**a**), generalized clonus (**b**) and forelimb tonus (**c**) in the i.v. PTZ seizure threshold test in mice. α-Spinasterol was administered i.p. 30 min prior to the test. The doses are shown on the abscissa. Control animals received 1 % DMSO. Each bar represents the mean (mg/kg PTZ) + SEM. *p < 0.05; ***p < 0.001 vs. the control group (one-way ANOVA followed by Dunnett’s post hoc test)

The effect of α-spinasterol on the threshold for the first myoclonic twitch is shown in Fig. 1a [one-way ANOVA: *F*(4,63) = 13.560, *p* < 0.0001]. α-Spinasterol at a dose of 0.1 mg/kg slightly raised the threshold for the first myoclonic twitch (*p* < 0.05 vs. the vehicle-treated group). Higher doses, 0.5 and 1 mg/kg, caused further increase in the seizure threshold (*p* < 0.001 vs. the control group). The lowest dose of 0.02 mg/kg was ineffective.

The effect of α-spinasterol on the threshold for the generalized clonus with loss of righting reflex is shown in Fig. 1b [one-way ANOVA: *F*(4,63) = 3.412, *p* = 0.014]. α-Spinasterol at doses of 0.02 and 0.1 mg/kg had no effect on the seizure threshold. However, higher doses 0.5 and 1 mg/kg significantly raised the thresholds for the onset of clonic seizures as compared to the control group (*p* < 0.05).

Figure 1c presents the influence of α-spinasterol on the threshold for the forelimb tonus (one-way ANOVA: *F*(4,60) = 1.991, *p* = 0.107). α-Spinasterol at any doses tested did not affect the susceptibility of mice to the PTZ-induced forelimb tonus.

### Effects of α-spinasterol in the MEST test

**Fig. 2:**
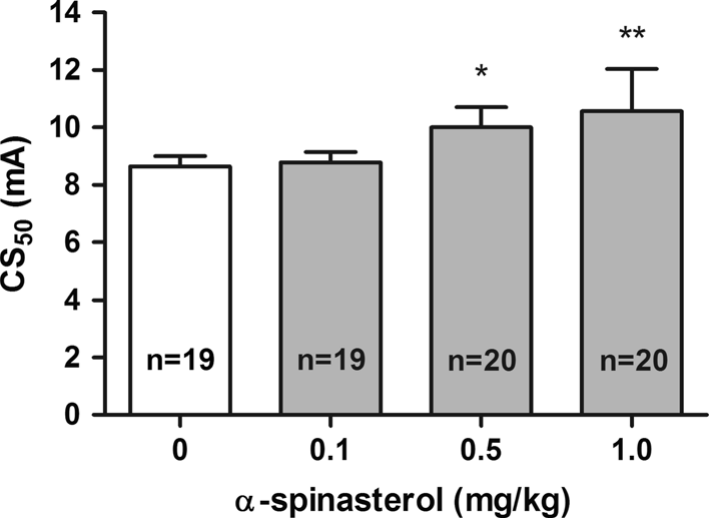
Effect of α-spinasterol on the seizure threshold in the MEST test. α-Spinasterol was administered i.p. 30 min prior to the test. The doses are shown on the abscissa. Control animals received 1 % DMSO. Data are presented as CS_50_ (in mA) values with upper 95 % confidence limits. Each CS_50_ value represents current intensity predicted to produce convulsions in 50 % of mice. *p < 0.05; **p < 0.01 vs. the control group (one-way ANOVA followed by Dunnett’s post hoc test)

The influence of α-spinasterol on the threshold for the tonic hindlimb extension in the MEST test is shown in Fig. 2 [one-way ANOVA: *F*(3,34) = 5.752, *p* = 0.0027]. α-Spinasterol at a dose of 0.1 mg/kg did not affect the seizure threshold. Post hoc analysis revealed statistically significant increase in the CS_50_ value for doses of 0.5 and 1 mg/kg (*p* < 0.05 and *p* < 0.01 vs. the vehicle-treated group, respectively).

### Effects of α-spinasterol in the 6 Hz seizure test

**Fig. 3:**
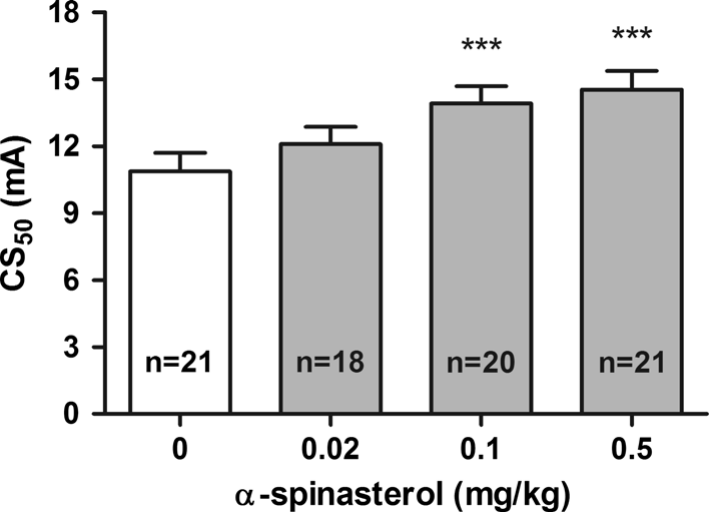
Effect of α-spinasterol on the seizure threshold in the 6 Hz seizure test. α-Spinasterol was administered i.p. 30 min prior to the test. The doses are shown on the abscissa. Control animals received 1 % DMSO. Data are presented as CS_50_ (in mA) values with upper 95 % confidence limits. Each CS_50_ value represents current intensity predicted to produce convulsions in 50 % of mice. ***p < 0.001 vs. the control group (one-way ANOVA followed by Dunnett’s post hoc test)

The effect of α-spinasterol on the threshold for the 6 Hz-induced psychomotor seizures is shown in Fig. 3 [one-way ANOVA: *F*(3,33) = 19.000, *p* < 0.0001]. The threshold for the 6 Hz-induced seizures was not affected by α-spinasterol injected at a dose of 0.02 mg/kg. At higher doses (0.1 and 0.5 mg/kg), α-spinasterol significantly increased the CS_50_ values as compared to the control group (*p* < 0.001).

### Effect of α-spinasterol on the neuromuscular strength, motor coordination and body temperature

Table 1 presents the influence of α-spinasterol at doses ranging from 0.02 to 1 mg/kg on the neuromuscular strength, motor coordination and rectal temperature changes. α-Spinasterol at any doses tested did not affect significantly the neuromuscular strength in mice, as determined in the grip-strength test [one-way ANOVA: *F*(4,55) = 0.890, *p* = 0.476]. Likewise, there was no significant impairment of motor coordination in the chimney test (Fisher’s exact test: *p* > 0.05). Furthermore, α-spinasterol did not change significantly body temperature, as determined by comparison of the differences between the pre-injection and post-injection values of rectal temperature in mice [one-way ANOVA: *F*(4,64) = 1.910, *p* = 0.120].

**Table 1:** Effect of α-spinasterol on neuromuscular strength, motor coordination and rectal temperature in mice

Treatment	Neuromuscular strength (mN/g)	*n*	Impairment of motor coordination (%)	*n*	Δ*T* (°C)	*n*
1 % DMSO	32.45 ± 1.55	12	0	12	+0.45 ± 0.15	11
α-Spinasterol 0.02 mg/kg	29.73 ± 1.43	12	0	12	+0.22 ± 0.15	12
α-Spinasterol 0.1 mg/kg	30.03 ± 1.33	12	8.33	12	+0.09 ± 0.17	12
α-Spinasterol 0.5 mg/kg	28.88 ± 1.49	12	0	12	+0.24 ± 0.15	11
α-Spinasterol 1 mg/kg	30.58 ± 1.26	12	0	12	+0.69 ± 0.22	12

Data are presented as mean ± SEM grip strengths in millinewtons per gram of mouse body weight (mN/g) from the grip-strength test, assessing skeletal muscular strength in mice, as a percentage of animals showing motor coordination impairment in the chimney test and as the differences between the pre-injection and post-injection values of rectal temperature in mice (Δ*T*)

## Discussion

Although the TRPV1 receptors are widely distributed thought the brain, still little is known about their function in the central nervous system (Martins et al. 2014). Recently, the TRPV1 receptors have been proposed to participate in pathogenesis of epilepsy (Fu et al. 2009; Gonzalez-Reyes et al. 2013; Shirazi et al. 2014). In the present study, we aimed to provide more data on the role of TRPV1 antagonism in the seizure threshold control.

Our findings showed that α-spinasterol, a plant-derived compound that was reported to act as a TRPV1 antagonist, elevates the seizure threshold in three acute seizure threshold tests in mice. In the i.v. PTZ seizure threshold test, α-spinasterol increased the seizure threshold for the onset of both the first myoclonic twitch and the generalized clonic seizures but had no significant effect on the PTZ-induced tonic forelimb extension. In contrast, α-spinasterol significantly raised the seizure threshold for the tonic hindlimb extension in the MEST test. The discrepancy between the effect of α-spinasterol on tonic seizures in the i.v. PTZ test and the MEST test could have been a consequence of distinct mechanisms underlying seizure induction in these two tests. The i.v. PTZ seizure test employed in the present study models myoclonic and generalized tonic–clonic seizures (White 1998). Since PTZ is a blocker of the picrotoxin site of the chloride ionophore of the GABA_A_ receptor complex, the PTZ seizure threshold is particularly sensitive to drugs that affect GABAergic neurotransmission (Löscher 2009). The MEST test is considered to be a model of generalized tonic–clonic (grand mal) seizures in humans and it can be useful for detecting anticonvulsant agents that act as sodium channel blockers (Castel-Branco et al. 2009; Nieoczym et al. 2014). In addition, our results demonstrated that α-spinasterol significantly increased the threshold in the 6 Hz psychomotor seizure threshold test that is considered to be a model of therapy-resistant limbic seizures (Barton et al. 2001).

The anticonvulsant effect of α-spinasterol might be a consequence of the inhibition of glutamate release. Glutamate is a major excitatory neurotransmitter that is responsible for the initiation and spread of seizure activity. Activation of the TRPV1 receptors leads to the increased Na^+^ and Ca^2+^ ions influx and depolarization of neurons (Manna and Umathe 2012b). It was reported that stimulation of TRPV1 receptors increases the release of glutamate which in turn may affect the release of other neurotransmitters such as dopamine and GABA (Martins et al. 2014). Thus, the TRPV1 receptors appear to play a role in the regulation of neuronal excitability. For example, Mori et al. (2012) demonstrated that the TRPV1 receptors regulate cortical excitability in humans through modulation of glutamate release. Activation of the TRPV1 receptors with capsaicin or anandamide was also shown to induce neuronal death both in vitro and in vivo, which suggests a possible neuroprotective action of the TRPV1 receptor antagonists. Furthermore, the TRPV1 receptors were reported to play a role in hippocampal synaptic plasticity by facilitating long-term potentiation and suppressing long-term depression. These effects may be important for seizure activity (Martins et al. 2014; Vilela et al. 2014). Hippocampus is a possible site of the anticonvulsant action of α-spinasterol since it had cytoprotective effects against glutamate-induced neurotoxicity in mouse hippocampal HT22 cell (Jeong et al. 2010). However, this issue needs further investigation.

It is widely accepted that the immune system is involved in epileptogenesis (Li et al. 2011). For that reason, anti-inflammatory properties of α-spinasterol might have contributed to its anticonvulsant effects. α-Spinasterol suppressed production of pro-inflammatory cytokines and inflammatory mediators in the lipopolysaccharide-induced inflammation in mice (Borges et al. 2014) as well as in the BV2 microglia cells (Jeong et al. 2010). Noteworthy, α-spinasterol was also reported to reduce the expression of the inducible nitric oxide (NO) synthase, thereby decreasing NO production. It should be mentioned here that NO oxide is a multifunctional signaling molecule but it is also a key mediator of excitotoxic neuronal injury and it is involved in neurobiology of numerous diseases including epilepsy (Rundfeldt et al. 1995; Nieoczym et al. 2012).

α-Spinasterol has been chosen for our studies because of its aforementioned ability to cross the blood–brain barrier and low toxicity (Trevisan et al. 2012). It exerted anticonvulsant effects in mice at small doses of 0.02–1 mg/kg (i.p. route of administration), whereas the lethal dose (LD_50_) of α-spinasterol after i.p. injection in mice was estimated at 479 mg/kg (Zhou et al. 1985). In our study, α-spinasterol produced no acute adverse effects. It did not affect motor coordination or neuromuscular strength, as assessed in the chimney and the grip-strength test, respectively. Moreover, no signs of hyperthermia at any dose used were observed, which is in line with the study of Trevisan et al. (2012). Altogether, α-spinasterol seems to be a useful tool for studying the role of central TRPV1 receptors in animal studies and perhaps in human subjects on condition that the ability to block the TRPV1 receptors is the main mode of action of α-spinasterol.

It is widely known that the pharmacological blockade of the TRPV1 receptors elicits marked hyperthermia. The lack of hyperthermic response after α-spinasterol administration may suggest that the compound acts in other manners that exert antipyretic effects. However, several hyperthermia-free TRPV1 antagonists have been already developed (Lehto et al. 2008; Garami et al. 2010; Watabiki et al. 2011; Andreev et al. 2013; Voight et al. 2014). Furthermore, Garami et al. (2010) reported that the hypothermia induced by TRPV1 antagonists is triggered by the blockade of the proton mode of TRPV1 activation. They concluded that the hyperthermia-free TRPV1 antagonists do not block TRPV1 activation by protons and they work in different manner. Further studies on the pharmacological profile of α-spinasterol are required to elucidate its mode of action at the TRPV1 receptors.

It is also worth mentioning that α-spinasterol is a plant steroid. Natural remedies have drawn attention of researchers as a promising source of new biologically active compounds for years. A number of herbs used in folk medicine as well as single plant-derived compounds have been shown to possess anticonvulsant activity (Schachter 2009). Cannabinoids can be an example here (Devinsky et al. 2014). Interestingly, the TRPV1 receptors are often co-localized with cannabinoid CB_1_ receptors. Anandamide, an endogenous agonist of CB_1_ receptors, was shown to activate the TRPV1 receptors, therefore it exhibits both pro- (at lower doses) and anticonvulsant (at higher doses) effects by activating the TRPV1 and CB_1_ receptors, respectively (Manna and Umathe 2012b). We can speculate that the endocannabinoid system may be also somehow involved in the anticonvulsant effect of α-spinasterol but this suggestion should be carefully evaluated in other studies.

In conclusion, the present study demonstrates for the first time that α-spinasterol, a TRPV1 receptor antagonist, exerts acute anticonvulsant effects in three distinct seizure threshold tests in mice. Our findings add support to the thesis concerning suppression of ongoing seizures by TRPV1 antagonists. α-Spinasterol may represent a new pharmacological approach towards the development of novel or adjuvant therapy for seizure disorders. Nevertheless, further detailed studies on the anticonvulsant action of α-spinasterol in other animal models of seizures and epilepsy are required. Its possible interactions with antiepileptic drugs should be also evaluated.

## Abbreviations

CB_1_: Receptor, cannabinoid type 1 receptor
CL: Confidence limit
CS_50_: Current strength required to induce seizure response in 50 % of mice
DMSO: Dimethyl sulfoxide
i.p.: Intraperitoneally
i.v.: Intravenous
MEST: Maximal electroshock seizure threshold
NO: Nitric oxide
PTZ: Pentylenetetrazole
SEM: Standard error of the mean
TRPV1: Transient receptor potential vanilloid 1 receptor
